# Postpartum Depression: Epidemiological Clinical Profile of Patients Attended In a Reference Public Maternity in Salvador-BA

**DOI:** 10.1055/s-0038-1676861

**Published:** 2019-03-01

**Authors:** Ivan de Sousa Araújo, Karolina Sales Aquino, Luciana Kelly Amado Fagundes, Vanessa Cruz Santos

**Affiliations:** 1 Faculty of Medicine, Universidade Salvador, Salvador, BA, Brazil; 2 Institute of Collective Health, Universidade Federal da Bahia, Salvador, BA, Brazil

**Keywords:** postpartum depression, clinical profile, epidemiological profile, prevalence, risk factors, depressão pós-parto, perfil clínico, perfil epidemiológico, prevalência, fatores de risco

## Abstract

**Objective** To evaluate the clinical epidemiological state of women with suspected postpartum depression (PPD) in a public maternity hospital in Salvador, state of Bahia, Brazil.

**Methods** A cross-sectional research was performed with puerperal patients attended at a public maternity hospital in Salvador, Bahia. Data collection was performed from June to September 2017. The Edinburgh Postnatal Depression Scale was used as a screening instrument, and, subsequently, women with positive scores answered a questionnaire to identify their clinical and epidemiological status.

**Results** Out of 151 postpartum women from the research, 30 (19.8%) presented suspicion of PPD. There was a prevalence of single mothers 13 (43.3%), women with complete fundamental education 15 (50.0%), women with black skin color 14 (46.7%), and those with a monthly family income of up to one minimum wage 18 (40.0%).

**Conclusion** Although PPD is an underdiagnosed disease, a high prevalence of the condition was found in our research. It is, then, considered that these results reinforce its significance as a public health problem, requiring prevention strategies, early diagnosis and effective treatment.

## Abstract (pt)

**Resumo:**

**Objetivo** Avaliar o perfil clínico epidemiológico de mulheres com suspeita de Depressão Pós-Parto em uma maternidade pública de referência de Salvador, no estado da BA.

**Métodos** Estudo transversal, realizado com puérperas atendidas em uma maternidade pública de referência de Salvador, BA. A coleta de dados foi realizada de junho até setembro de 2017. Utilizou-sea escala de Edimburgo como instrumento, e, posteriormente, as mulheres com escore positivo responderam a um questionário para a identificação do seu perfil clínico e epidemiológico.

**Resultados** Das 151 puérperas pesquisadas, 30 (19,8%) apresentaram suspeita de depressão pós-parto. Predominaram as puérperas solteiras 13 (43,3%), com ensino médio completo 15 (50,0%), cor da pele preta 14 (46,7%), e aquelas com renda familiar mensal de até um salário mínimo 18 (40,0%).

**Conclusão** Ainda que a depressão pós-parto seja uma enfermidade subdiagnosticada, neste estudo verificou-se uma elevada prevalência da condição. Considera-se, então que estes resultados reforçam o seu significado como problema de saúde pública, exigindo estratégias de prevenção, diagnóstico precoce e tratamento efetivo.

## Introduction

Postpartum depression (PPD) refers to a set of symptoms that includes mood, cognitive, psychomotor, and vegetative changes. Postpartum depression usually starts between the fourth and eighth week after delivery, a time marked by hormonal and social changes in family organization and female identity.^1^ The prevalence of PPD worldwide is 5 to 20%, while in Brazil it is between 12 and 37%.^2^ ^3^ ^4^

In the puerperium, abrupt changes in the levels of thyroid and gonadal hormones, oxytocin levels and the hypothalamic-pituitary-adrenal axis occur. In addition to biological changes, maternity is marked by important psychological, social, sexual and financial changes.^3^ ^5^ ^6^ The combination of these factors in the postpartum period characterizes the puerperium as a period of great vulnerability for the appearance of psychiatric disorders, such as baby blues and puerperal psychoses, or for the precipitation of anxiety disorders.^6^

In puerperal mood disorders, we have two other categories in addition to postpartum depression: baby blues (maternity blues, melancholy of motherhood or puerperal dysphoria) and puerperal psychoses.^1^ ^3^ ^6^

Baby blues are characterized by milder depression, reaching ∼ 60% of new mothers between the 3^rd^ and 5^th^ day postpartum, and usually have spontaneous remission. The clinical picture includes easy crying, affective lability, irritability, and hostile behavior with family and companions. Women with puerperal dysphoria do not require pharmacological intervention; the approach is designed to provide adequate emotional support, understanding and help in caring for the baby.^1^ ^3^ ^6^

Puerperal psychosis is the most serious mental disorder that can occur in the puerperium and is a risk situation for the occurrence of suicide or infanticide. It has a prevalence ranging from 0.2 to 1%, and its onset is usually rapid. Psychotic and affective symptoms settle in the early days, and include euphoria, irritable mood, logorrhea, agitation and insomnia, evolving with delusions, persecutory ideas, hallucinations, and disorganized behavior. Infanticide usually occurs when delusional ideas involve the baby. Since the picture of puerperal psychosis is severe, hospitalization is usually required.^1^ ^3^ ^6^

Anxiety disorders may also be exacerbated or precipitated in the puerperium, especially generalized anxiety disorders, posttraumatic stress disorder and obsessive-compulsive disorder.^3^

The factors strongly associated with PPD are: personal history of depression, depressive or anxious during pregnancy, stressful life events, poor social and financial support, and conflicting marital relationships. Other likely risk factors are family history of psychiatric disorders, previous maternity blues episode, low level of schooling, and low self-esteem. Obstetric complications, premature delivery, difficulty in breastfeeding, cultural factors, history of sexual abuse or unwanted pregnancies are also associated.^1^ ^2^ ^3^ ^7^ ^8^ ^9^ ^10^

The puerperal depressive disorders affect the mother-child binomial, causing serious changes in the psychosocial and family dynamics, with significant impairment in the structure of the psyche of the child.^7^ ^11^ ^12^ ^13^ ^14^ The figure that should be nurturing becomes the figure that neglects and does not provide the child with the demands that the age requires. In addition, these disorders cause weariness in the relationship of the puerpera with her relatives and with her partner, and increase the possibility of auto and heteroaggression.^1^ ^2^ ^3^ ^15^

The diagnosis of PPD is not always easy and unequivocal, since the clinical picture can be varied in the presentation and intensity of the symptoms and can be neglected by the puerperium itself and by its relatives.^8^ ^9^ As one of the diagnostic tools, there is the Postpartum Depression Self-Assessment Scale. It is a scale with the presence and intensity of depressive symptoms in the 7 days prior to its application, which is quick and simple to use, and has high sensitivity and specificity.^2^ ^8^ ^10^ ^16^ ^17^ ^18^ ^19^

There are few data on the disease/age or status of patients with PPD in Salvador. Furthermore, it is known that less than 25% of postpartum women have access to treatment, and only 50% of PPD cases are diagnosed during clinical exercise.^10^ These facts emphasize the importance of approaching the theme, and of reassessing the need to expand the horizon of research in this area in Salvador, state of Bahia.

This study aims to evaluate the epidemiological clinical profile of patients with suspected PPD in a reference public maternity hospital in Salvador, Bahia, from June to September 2017, to determine the prevalence of PPD in the patients studied; to describe the frequency of factors associated with the development of PPD, and to evaluate the association between social determinants and PPD in the patients studied.

## Methods

This research is a cross-sectional study performed with 151 postpartum women attended at a reference maternity hospital in Salvador, state of Bahia (Maternidade Climério de Oliveira [MCO, in the Portuguese acronym]).

Data collection took place through the application of two instruments: the first was the Postpartum Depression Self-Assessment Scale or Edinburgh Score—it contains 10 questions, each with a maximum score of 3 points (Appendix A). The second was a questionnaire to evaluate clinical, social and economic variables, applied only to postpartum women who reached a score of 10 or more (meaning possible depression) (Appendix B). The questionnaires aimed at selecting women with suspected PPD and to draw the clinical and epidemiological profile of the latter.

**Appendix A:**
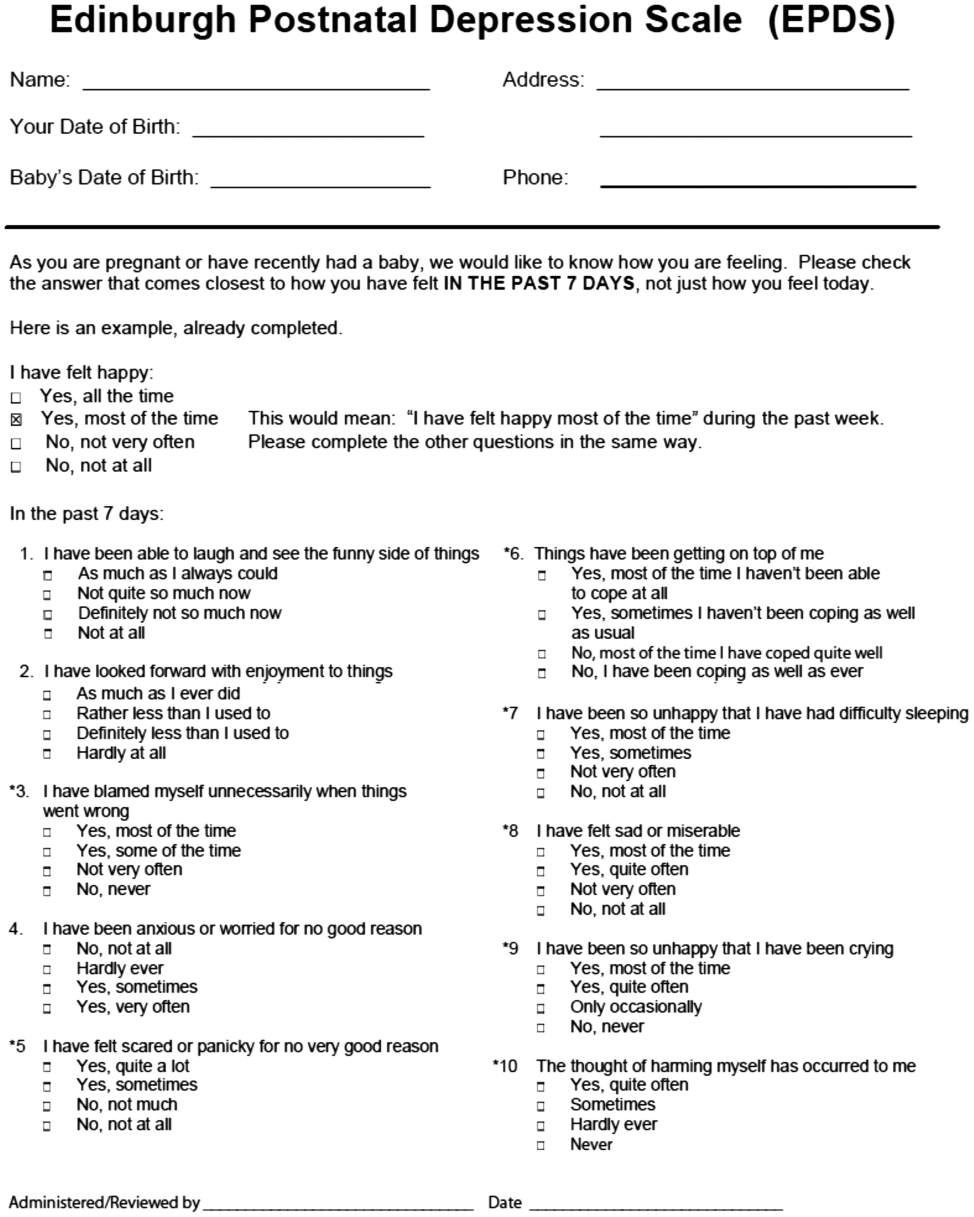
Edinburgh Postnatal Depression Scale.

**Appendix B:**
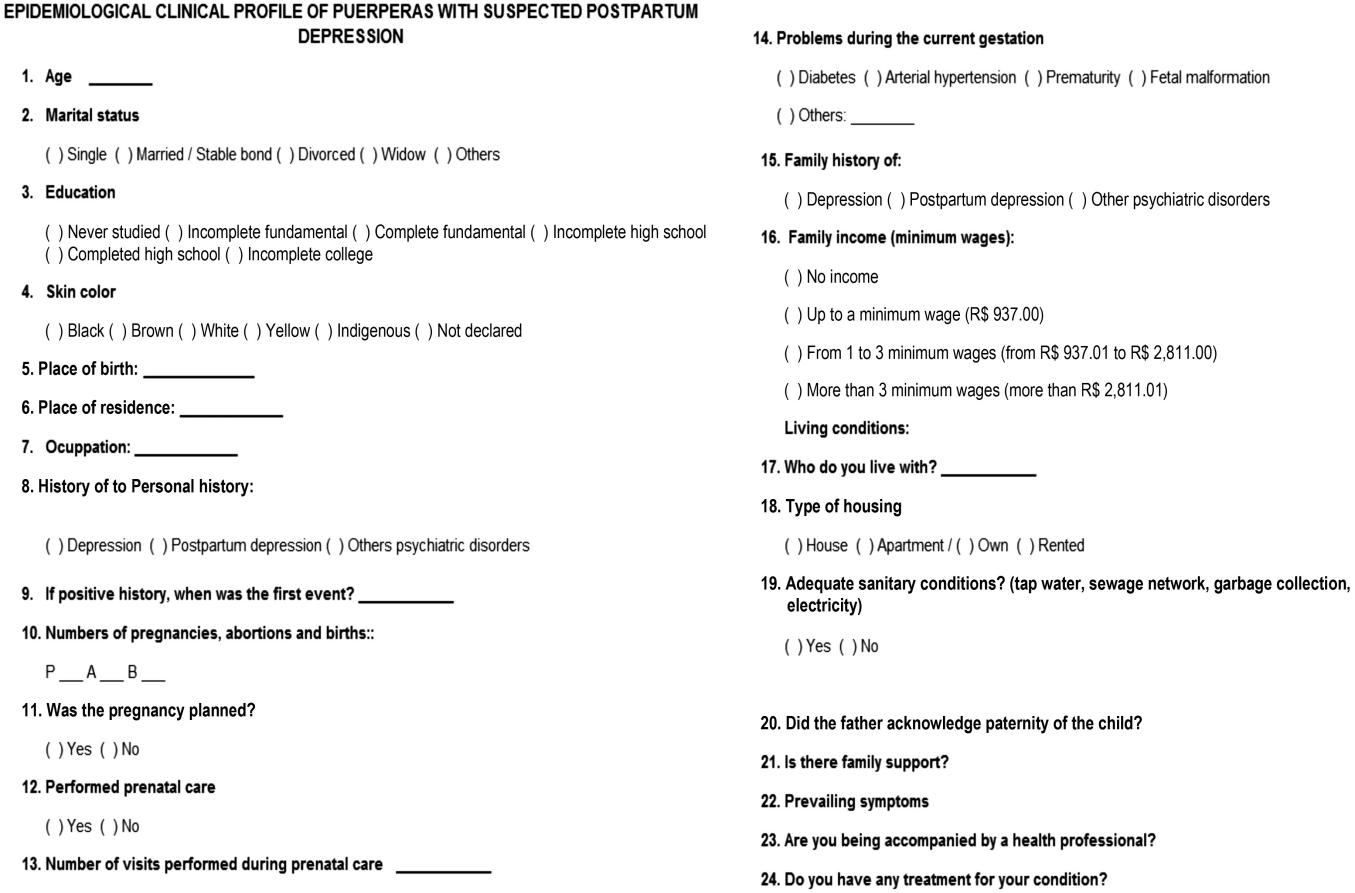
Questionnaire on clinical and epidemiological characteristics.

For the clinical state, we analyzed: the most prevalent symptoms, personal history (history of depression or other psychiatric disorders) and the date of the first event (if any), family history, comorbidities (diabetes, hypertension, etc.) or complications in relation to the baby (prematurity, malformations etc). For the epidemiological profile, we analyzed: age, ethnicity/color, place of birth and origin, marital status, educational level, occupation, pregnancy (planned/unplanned), number of pregnancies, abortion or not, family income, living conditions and psychosocial conditions (did the father acknowledge paternity of the child? Is there family support?)

The women who had recently given birth were approached in the ward units of the MCO. At the initial approach, the Term of Free and Informed Consent or the Term of Assent for children under 18 years were applied. In that first moment, the researchers got the phone numbers of the participants, explaining that they would be contacted by telephone within a period from 4 to 8 weeks for the application of the questionnaires.

Faced with the fact that most symptoms of PPD occur within 4 to 8 weeks postpartum, it is clear that this is the time interval to undergo the application of the questionnaires so that they are the more reliable.

The measure was of convenience, and women who delivered their children at the MCO between May and September 2017 were included. The women who delivered outside the delimited time period were excluded from the study. The dates were tabulated and analyzed by Excel Microsoft XP (Microsoft Corp., Redmond, WA, USA), through tables, as this is a quantitative research, in addition to having subsidies to the pertinent literature.

All postpartum women with suspected PPD (those who scored ≥ 10 on the Edinburgh Scale) were sent to the psychiatry services of the MCO, where they were cared for and given all the necessary care.

This study complies with Resolution No. 466/2012 of the National Health Council and was approved by the Research Ethics Committee of the Universidade Salvador with the participation of the MCO under protocol no. 2,087,464 and CAAE n° 64729517.0.0000.5033.

## Results

Based on the evaluation of the 151 postpartum women attended at the MCO, 30 women were identified for suspected PPD, which means a prevalence of 19.8%.

Among the 30 postpartum women, the ages varied from 15 to 40 years (average of = 24.43 years), with a higher percentage for the age group from 20 to 24 (46.7Single mothers (13 ; 43.3%), women with complete fundamental education (15 ; 50.0%), those with black skin color (14 ; 46.7%), those born in Salvador, BA (18 (; 60%), those residing in Salvador-BA 25 (83; 3%), housewives (15 ; 50%), women with an average income of up to a minimum wage (18 ; 60%), women living with husband/partner and children (15 ; 50%), and those living in their own home (15 ; 50.0%) were more prevalent (Table 1).

**Table 1:** Sociodemographic characteristics of puerperal women with suspected postpartum depression (PPD)

Characteristics	n (%)
**Age group (years)**	
15–19	05 (16.7)
20–24	14 (46.7)
25–40	11 (36.7)
**Marital status**	
Single	13 (43.3)
Stable bond	12 (40.0)
Married	05 (16.7)
**Education**	
Never studied	01 (3.3)
Incomplete fundamental	04 (13.3)
Complete fundamental	02 (6.7)
Incomplete high school	07 (23.3)
Complete high school	15 (50.0)
Incomplete college	01 (3.3)
**Skin color**	
Black	14 (46.7)
Brown	13 (43.3)
White	02 (6.7)
Indigenous	01 (3.3)
**Place of birth**	
Salvador, BA	18 (60.0)
Cities in the countryside of Bahia	09 (30.0)
Cities in other Brazilian states	02 (6.7)
Cities in other countries	01 (3.3)
**Place of residence**	
Salvador, BA	25 (83.3)
Cities in the countryside of Bahia	05 (16.7)
**Occupation**	
Housewife	15 (50.0)
Unemployed	04 (13.3)
Student	04 (13.3)
Saleswoman	02 (6.6)
Bar owner	01 (3.3)
Nanny	01 (3.3)
Manicurist	01 (3.3)
Cash operator	01 (3.3)
Telemarketing clerk	01 (3.3)
* **Monthly family income (minimum wages)**	
Up to 1	18 (60.0)
From 1–3	11 (36.7)
** NI	01 (33.3)
**Lives with**	
Spouse/partner and children	15 (50.0)
Children (only)	02 (6.6)
Others (father, mother, brother/sister, in-laws, brother or sister-in-law, friends)	13 (43.3)
**Place of residence**	
Own house	15 (50.0)
Rented house	08 (26.7)
Own apartment	03 (10.0)
Rented apartment	04 (13.3)

* Minimum salary in force = R$ 937.00.

** NI = Not informed.

Regarding the clinical characteristics of postpartum women, there was a higher prevalence of those with only one pregnancy 14 (46.7%), one childbirth 17 (56.7%), no abortion 22 (73.3%), unplanned pregnancy 24 (80%), who had undergone prenatal consultation 30 (100%), and of these, 21 (70%) had ≥ 6.

During the current gestation, 19 postpartum women reported comorbidities (63.3%), some related to themselves, such as: deep sadness, toxoplasmosis, gestational hypertension, isthmus-cervical insufficiency, uterine myoma, gestational diabetes, HIV virus complications; and others related to the newborn: prematurity, jaundice, convulsion, cardiac anomaly and death.

Only 5 postpartum women had a personal history of psychiatric disorders (16.7%) and 10 reported a family history of psychiatric disorder (33.3%), with depression prevailing in 9 of them (90%). Of all the patients analyzed, only 1 (3.3%) was being followed up by a mental health professional and being treated for her condition (Table 2).

**Table 2:** Clinical characteristics of puerperal women with suspected postpartum depression (PPD)

Characteristics	n (%)
**Number of pregnancies**	
1	14 (46.7)
2–4	13 (43.3)
From 5–7	03 (10.0)
**Number of births**	
1	17 (56.7)
2–4	12 (40.0)
5–7	01 (3.3)
**Number of abortions**	
0	22 (73.3)
1	04 (13.3)
2–4	04 (13.3)
**Was the pregnancy planned?**	
Yes	06 (20.0)
No	24 (80.0)
**Did you attend to prenatal care visits?**	
Yes	30 (100.0)
No	0 (0)
**Number of visits performed during prenatal care**	
1–5	09 (30.0)
≥ 6	21 (70.0)
**Did you have problems during the current gestation?**	
Yes	19 (63.3)
No	11 (36.7)
**Do you have a personal history of psychiatric disorders?**	
Yes	05 (16.7)
No	25 (83.3)
**Do you have a family history of psychiatric disorders?**	
Yes	10 (33.3)
No	20 (66.7)

Regarding the psychosocial factors, there was a predominance of postpartum women who reported having the father of the child present 24 (80%), receiving family support 17 (56.7%) and presenting prevailing health-related symptoms during pregnancy and puerperium mental illness 28 (93.3%). Among these symptoms, sadness (33.3%) and tiredness (10%) predominated (Table 3).

**Table 3:** Psychosocial characteristics of postpartum women with suspected postpartum depression (PPD)

Characteristics	n (%)
**Is the father present? (acknowledged paternity of the child)**	
Yes	24 (80.0)
No	06 (20.0)
**Is there family support?**	
Yes	17 (56.7)
No	09 (30.0)
More or less	04 (13.3)
**Are there any prevalent symptoms related to mental health?**	
Yes	28 (93.3)
No	02 (6.7)

## Discussion

There are many risk factors, besides genetic predisposition, associated with PPD, such as socioeconomic and epidemiological factors, which make PPD a multifactorial condition. Although its etiology is not clearly known, some factors may contribute to the precipitation of PPD, among which the following are cited: low socioeconomic status; non-acceptance of pregnancy; greater number of pregnancies, previous childbirths and living children; shorter relationship time with partner; history of obstetric problems; longer skin-to-skin contact with the baby after birth; domestic violence; little support from the partner; overloading tasks; and conflicting experience of motherhood.^1^ ^2^ ^20^ ^21^ ^22^

The prevalence of PPD found in this study (19.8%) was within the numbers often found in the literature, which range from 10 to 20% of women, and can start within the 1^st^ week after delivery and last up to 2 years.^1^ The prevalence of PPD was high in this study, in agreement with other Brazilian studies, which makes it a public health problem in the country.

In this research, 46.6% of postpartum women had not finished high school or had not even started it, and 50% had completed high school. Added to this, there is the fact that only 1 mother (3.3%) had higher education. These education standards are in agreement with other analyzed studies and corroborate the idea that low educational level may contribute to the development of PPD.^22^ ^23^ ^24^

Some studies still provide a view between age and PPD: younger mothers presented depressive symptoms more frequently. The present study did not verify statistical relevance related to the age of the mother, since out of the 30 patients with probable PPD, the majority (14) were between 20 and 24 years of age, 11 were between 25 and 40 years old and only 5 patients were between the ages of 15 and 19, considered at greater risk.^18^ ^25^

Several studies point out as an important risk factor for the development of PPD an unplanned or unwanted pregnancy. In the present study, 80% of the deliveries were not planned, which shows the statistical relevance of this factor.^17^ ^23^ ^26^ ^27^ ^28^ ^29^

It was also verified that the majority of the postpartum women that had a precarious socioeconomic status were more susceptible to the development of PPD, since other researches affirm that PPD is influenced by poverty-related difficulties.^17^ ^30^ ^31^ ^32^ In the present study, 60% of the patients had less than 1 minimum wage as family income, and 36.7% had 2 to 3 minimum wages. According to the classification of the Brazilian Institute of Geography and Statistics (IBGE, in the Portuguese acronym), the sample participants fall into the lowest social classes, those being classes D and E.

Regarding marital status, this research shows a higher prevalence of single mothers, followed by mothers who are in stable union. Some studies suggest that among the main risk factors for changes in the postpartum period are the “single” or “divorced” marital status.^27^ ^33^ ^34^ In other Brazilian studies, there was a predominance of stable union as marital status. This type of union is characterized mainly by its instability, which can provoke frequent conjugal conflicts, contributing to the development of depressive maternal symptomatology, and even favoring carelessness with the baby.^20^ In addition, it is important to investigate the quality of marital ties and not exclusively the presence or absence of a partner.^35^

Within the context of PPD, the importance of understanding and family support in the postpartum period is emphasized, so that the mother knows that there is nothing wrong with her. Being accepted as a mother helps a lot to decrease the malaise, contributing to the recovery from PPD.^7^ It contributes to the development of this picture that the expectations placed on women at the moment are unrealistic: an idealized pattern of mother as a “competent caregiver,” always controlled, loving unconditionally, being able to handle domestic tasks, baby care, full-time employment and still meet the demands of the partner. When the woman realizes that she cannot handle all the demands she has, feelings of sadness, anger, guilt, anxiety, and depression can become present.^36^

Another important category is the complications experienced by women during pregnancy or with their newborn babies after childbirth. These complications are considered as precipitants for maternal depression.^11^ ^21^ ^24^ ^32^ ^36^ In this research, most women with suspected PPD presented some type of problem that required medical care, such as gestational hypertension, isthmus-cervical insufficiency, gestational diabetes; and others related to the newborn, such as prematurity, jaundice, convulsion, cardiac anomaly and death. The relationship between obstetric complications and PPD is controversial, so there are studies showing positive and negative associations.

In addition to the factors highlighted above, several studies have revealed that previous history of psychiatric illness or previous psychological problem of the mother, including the melancholy of motherhood, also predicted the subsequent occurrence of PPD.^24^ ^25^ ^37^ ^38^ ^39^

The high prevalence of PPD nowadays reinforces its significance as a public health problem, requiring prevention and treatment strategies.^40^ ^41^ ^42^ A careful follow-up of mothers, especially those with low incomes, through integrated actions that take into account the variables associated with depression, can prevent serious personal and family problems that result from PPD.^15^

Although the experience of postpartum women who have depressive symptoms is still poorly explored, some studies investigating this theme have been consistent in showing that depressed mothers commonly report more difficulty in mothering than non-depressed mothers.^3^

## Conclusion

In this research, the following characteristics prevailed among postpartum women who had scores for suspected PPD: age ˂ 24 years, single civil status or stable union, low income, low level of schooling, unplanned pregnancy and/or complications with the newborn. The high prevalence of PPD reinforces its significance as a public health problem, requiring prevention and treatment strategies. Careful and professional monitoring of mothers, especially those with risk factors, should be performed to prevent personal and family problems arising due to PPD. Knowledge of risk factors prepares the health team for effective intervention and action, with more successful results. It is worth noticing that those women with lower socioeconomic status may be more susceptible to the development of PPD, and are more likely to be neglected in their diagnosis, since they have greater difficulty in accessing health care; prenatal care often represents the only opportunity for continued care for the women's health. In addition, knowledge of the signs and symptoms of PPD should be disseminated, so that affected women are promptly diagnosed and referred for appropriate treatment. The application of the Edinburgh Score, a simple and rapid scale, should be disseminated in the health network, since it is ideal for use in the clinical routine by professionals who are not specialized in the area of mental health, to track mothers who present with depressive symptoms, thus not burdening specialized services.
